# Outcomes of Symptomatic Extracranial Carotid Artery Stenting in Octogenarians: A Single-Center Retrospective Study on Restenosis, Risk Factors, and Complications

**DOI:** 10.3390/medicina61030519

**Published:** 2025-03-17

**Authors:** Özgür Zülfükar Ertuğrul, Fırat Karaaslan, Reşit Yılmaz, Mehmet Cudi Tuncer

**Affiliations:** 1 Department of Radiology, Gazi Yaşargil Training and Research Hospital, University of Health Sciences, Diyarbakır 21090, Turkey; stenoz@hotmail.com; 2 Department of Neurology, Gazi Yaşargil Training and Research Hospital, University of Health Sciences, Diyarbakır 21090, Turkey; drfrt1321@gmail.com (F.K.); dr.resityilmaz@gmail.com (R.Y.); 3 Department of Anatomy, Faculty of Medicine, Dicle University, Diyarbakir 21200, Turkey

**Keywords:** carotid artery, Doppler ultrasonography, stenting, restenosis, elderly patients

## Abstract

*Background and Objectives:* This study aimed to evaluate the 6-month restenosis rate, risk factors, and complications following carotid artery stenting (CAS) in patients aged 80 years and older, assessing the efficacy and safety of CAS in this population. *Materials and Methods:* Fifty-six patients aged ≥80 years with symptomatic extracranial carotid stenosis who underwent CAS between May 2023 and August 2024 were retrospectively analyzed. Follow-up at 6 months included Doppler ultrasonography to assess restenosis. Demographic, clinical, and procedure-related complications were recorded, and risk factors for in-stent restenosis were evaluated. *Results:* Among the patients, 42.9% were female (n = 24) and 57.1% were male (n = 32), with a mean age of 85.3 ± 4.40 years. The restenosis rate was 12.5%. Restenosis was significantly associated with smoking (*p* = 0.002), severe stenosis (*p* = 0.016), and advanced age (*p* = 0.045). The minor complication rate was 5.3%, and no major complications were observed. Smoking and advanced age were identified as independent risk factors for restenosis. *Conclusions:* CAS is a safe and effective treatment option for elderly patients. However, those with a history of smoking, advanced age, or severe stenosis are at an increased risk of restenosis. These findings provide valuable insights into the outcomes and safety of CAS in patients aged 80 and older.

## 1. Introduction

The increasing elderly population and rising life expectancy have influenced neurovascular operators, making them more inclined toward aggressive treatment strategies for patients aged 80 years and older. The growing number of elderly individuals is expected to lead to an increase in older patients presenting with extracranial carotid stenosis. The primary cause of internal carotid artery stenosis (ICAS) is atherosclerosis, with secondary predisposing factors including arteritis, arterial dissection, and cervical radiotherapy. ICAS is a major cause of cerebral ischemic events and is more common in elderly individuals [1,2]. When the degree of ICAS exceeds 50%, the risk of cerebral ischemic events increases [3]. Restoring blood flow in extracranial carotid stenosis is critical for preventing stroke-related morbidity and mortality. Therefore, evaluating the therapeutic effects of ICAS from a surgical treatment perspective is essential for optimizing disease management [4,5].

Carotid artery stenting has been shown to not be less effective than carotid endarterectomy (CEA) and has been introduced as an alternative treatment for carotid stenosis [6,7]. CAS is a less invasive treatment method, particularly for high-risk patients. The most significant predictors of periprocedural ischemic events during CAS appear to be the presence of symptomatic stenosis, the absence of embolic protection devices, and advanced age [8]. These risks may be attributed to the elongation of the aortic arch and longer or more complex plaques, which increase the likelihood of complications during CAS [9,10]. Age-related systemic atherosclerosis and vascular anatomical changes associated with aging may also contribute to plaque formation. Consequently, the risk of stroke and mortality may be higher with CAS in elderly patients.

Some studies have raised concerns regarding whether CAS in elderly patients, particularly those in their eighties, is associated with excessive complication rates, potentially making CAS an unsuitable treatment option for this population [11]. In contrast, several single-institution studies that specifically examined the role of age in CAS outcomes reported no significant differences in adverse outcome rates between octogenarians and non-octogenarians [12,13]. Thus, uncertainty remains regarding the safety of CAS in octogenarians.

In this study, we analyzed the use of CAS and its perioperative outcomes in patients over 80 years of age with carotid artery stenosis in a single-center setting.

## 2. Material and Methods

### 2.1. Study Design and Patient Selection

**Figure 1:**
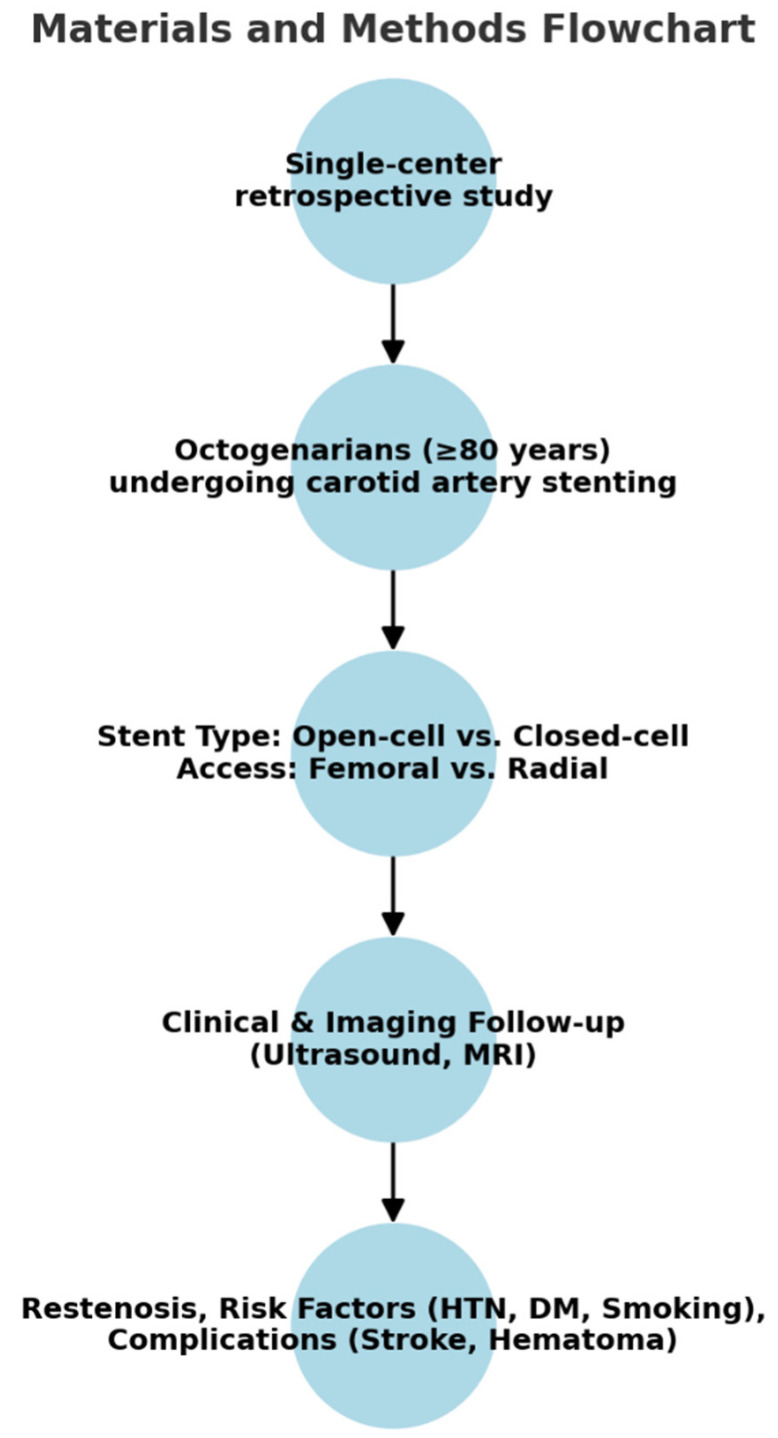
Flowchart illustrating the study design and methodology. The process begins with a single-center retrospective study on octogenarians (≥80 years) undergoing carotid artery stenting. Procedural details include the use of open-cell vs. closed-cell stents and femoral vs. radial access. Follow-up involves clinical and imaging assessments (ultrasound, MRI). Outcome assessment includes restenosis rates, risk factors (hypertension, diabetes, smoking), and complications such as stroke and hematoma.

This study is a single-center retrospective analysis evaluating the outcomes of CAS in octogenarians (≥80 years old). Patient data were collected from hospital records, including demographic characteristics, clinical history, procedural details, and follow-up findings (Figure 1). This study conformed to the principles of the 2013 Declaration of Helsinki and was approved by the Diyarbakır Gazi Yaşargil Training and Research Hospital Ethics Committee (No: 224/11 October 2024).

Hospital records of patients aged 80 years and older who underwent CAS for 50–99% symptomatic extracranial carotid stenosis between May 2023 and August 2024 were retrospectively analyzed.

Inclusion criteria:

- Patients aged ≥80 years.
- Symptomatic carotid artery stenosis (50–99%).
- History of transient ischemic attack (TIA) or ischemic stroke attributable to symptomatic extracranial carotid stenosis within the past six months.
- Underwent CAS during the study period.

Exclusion criteria:

- Incomplete follow-up data.
- History of a major cerebrovascular event (e.g., disabling stroke) prior to CAS.

### 2.2. Details of Stents Used

The stenting procedure was performed under standard interventional radiology protocols. Two primary types of stents were used:

- Open-cell stents (Wallstent, Boston Scientific, Natick, MA, USA);
- Closed-cell stents (Protégé RX carotid stent, Medtronic, Minneapolis, MN, USA).

The vascular access site for the procedure was one of the following:

- Femoral access, the preferred approach in most cases;
- Radial access, used in select cases based on patient anatomy and risk factors.

Procedural success was defined as successful stent deployment with residual stenosis <30% and no immediate complications.

### 2.3. Follow-Up and Assessment

All patients underwent clinical and imaging follow-up, including the following:

- Duplex ultrasonography at regular intervals;
- MRI or CT angiography, if restenosis was suspected.

Follow-up data focused on ISR (in-stent restenosis) occurrence, retreatment methods, and complications.

### 2.4. Outcome Measures and Analysis

The primary outcomes included the following:

(1) ISR rate based on imaging and clinical symptoms;
(2) Risk factors contributing to restenosis, such as hypertension, diabetes, and smoking history;
(3) Complications, including stroke, restenosis, hematoma, and other adverse events.

Patients with ISR were managed either with best medical therapy (BMT) or CAS reintervention.

This structured approach ensured comprehensive data collection and analysis, allowing for the assessment of restenosis rates, associated risk factors, and procedural safety in this elderly patient population.

### 2.5. Stenting Procedure and Perioperative Management

Before conventional angiography, all patients underwent noninvasive imaging, including CT or MR angiography or carotid Doppler ultrasonography. Written consent was obtained, and neurological examinations were conducted before and after angiography. Patients received 75 mg clopidogrel and 100 mg aspirin at least five days before stenting, continuing both for 3–6 months post stenting, followed by lifelong therapy with either clopidogrel or aspirin.

All procedures were performed under conscious sedation with anesthesiologist-administered analgesia. Distal embolic protection devices were used unless ICA anatomy or severe tortuosity prevented it. Patients received 3000–7000 U of unfractionated heparin before stenting, with self-expanding stents and post-dilation using 4.5–6.0 × 20 mm balloons. Atropine was given for bradycardia or hypotension, and manual compression was used for arterial closure.

Demographic data, comorbidities, stenosis severity, type 3 arch, contralateral stenosis, stent type, and access side were recorded. Patients were evaluated at discharge and at 1, 3, and 6 months. Doppler ultrasonography monitored stent patency and restenosis, documenting outcomes like death, major events, and restenosis, with treatments assessed at 6 months.

### 2.6. Statistical Analysis

Statistical analyses were conducted using SPSS 22 (IBM, Armonk, NY, USA). Continuous variables were presented as means ± standard deviations, and categorical variables as percentages. The Shapiro–Wilk test assessed distributions. Chi-square or Fisher’s exact tests compared categorical data, and *t*-tests or Mann–Whitney U tests analyzed continuous variables. Spearman’s rho evaluated associations, and multivariable logistic regression analyzed clinical parameters related to restenosis. Statistical significance was defined as *p* < 0.05.

## 3. Results

### 3.1. Patient Characteristics

Among 56 patients aged 80 and older, 42.9% were female (n = 24), and 57.1% were male (n = 32), with a mean age of 85.3 ± 4.40 years. Procedural success was 100%, and all patients were symptomatic. Moderate stenosis was observed in 42.9% of cases, while severe stenosis was found in 57.1% of cases. Open-cell stents were used in 33.9% of cases and closed-cell stents in 66.1%. Minor complications included periprocedural stroke, pseudoaneurysm, and femoral hematoma, each in 1.7% of cases. One patient experienced a minor stroke lasting less than 72 h, presenting with motor aphasia and frontal diffusion restriction on MRI. Restenosis occurred in 12.5% at six-month follow-up, with all patients remaining neurologically asymptomatic. Contralateral carotid artery occlusion was noted in 17.9%. Detailed data are in Table 1.

In this study, moderate stenosis was defined as a luminal narrowing of 50% to 69%, while severe stenosis was classified as 70% to 99%, based on the North American Symptomatic Carotid Endarterectomy Trial criteria. The degree of stenosis was measured using digital subtraction angiography, with the most stenotic segment compared to the normal distal arterial lumen. These definitions are widely accepted in cerebrovascular research and serve as standard thresholds for assessing carotid artery disease severity and guiding interventional decisions.

### 3.2. Significant Risk Factors for In-Stent Restenosis

**Figure 2:**
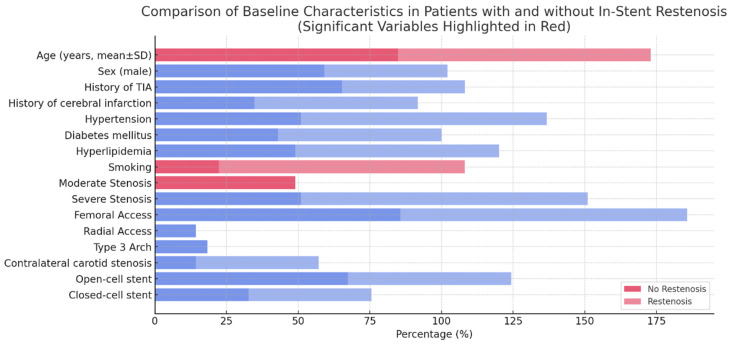
A horizontal bar chart comparing baseline characteristics in patients with and without in-stent restenosis. Statistically significant variables (*p* < 0.05) are highlighted in red, emphasizing their strong association with restenosis. Blue bars represent variables that are not statistically significant (*p* ≥ 0.05).

During the six-month follow-up, restenosis occurred in seven patients, who were divided into groups with and without restenosis. Advanced age, severe stenosis, and smoking were significantly associated with higher restenosis rates (Table 2, Figure 2).

A comparison of baseline characteristics between patients with and without in-stent restenosis revealed significant differences in several variables (Figure 2). Patients who developed in-stent restenosis were significantly older (mean ± SD) compared to those without restenosis. Additionally, smoking history and the presence of hyperlipidemia were more prevalent in the restenosis group. Regarding the severity of initial stenosis, moderate stenosis was observed more frequently in patients who later developed in-stent restenosis. However, severe stenosis showed no significant difference between the two groups. Sex distribution, history of TIA, history of cerebral infarction, hypertension, and diabetes mellitus did not exhibit significant differences between the restenosis and no-restenosis groups. Similarly, anatomical and procedural factors, such as femoral or radial access, the presence of a type 3 aortic arch, contralateral carotid stenosis, and stent design (open-cell vs. closed-cell), were comparable between the two groups.

These findings suggest that advanced age, smoking, and hyperlipidemia are important risk factors for in-stent restenosis, highlighting the need for targeted management strategies to mitigate restenosis risk in these patient populations.

### 3.3. Impact of Stent Type and Retreatment on ISR Grade

The choice between BMT and repeat CAS for treating ISR was primarily based on the severity of restenosis, in alignment with recommendations from the European Society for Vascular Surgery and other international guidelines. Patients with ISR ≤ 50–60% and without significant clinical symptoms were managed conservatively with BMT, including antiplatelet therapy, lipid-lowering agents, and strict risk factor modification. In contrast, patients with ISR ≥ 70%, particularly those showing progression or symptoms suggestive of hemodynamic compromise, underwent repeat CAS to restore adequate cerebral perfusion. This approach aligns with current best practices and reflects the individualized decision-making process for managing ISR in elderly patients.

Treatment included re-stenting for three patients and medical therapy for four. Open-cell stents were used in four cases and closed-cell stents in three, with no complications reported (Table 3). The analysis of ISR grade by retreatment type and stent design showed that ISR severity varied depending on both factors. Patients who underwent CAS exhibited a higher average ISR grade compared to those treated with best BMT. However, within each retreatment group, the ISR grade did not differ significantly between open-cell and closed-cell stents.

These findings suggest that while the choice of retreatment method influences ISR severity, the stent type may not play a decisive role in restenosis progression. Further investigations are needed to assess the long-term outcomes of different stent designs in ISR management.

### 3.4. Multivariate Analysis of Risk Factors for In-Stent Restenosis (ISR)

**Figure 3:**
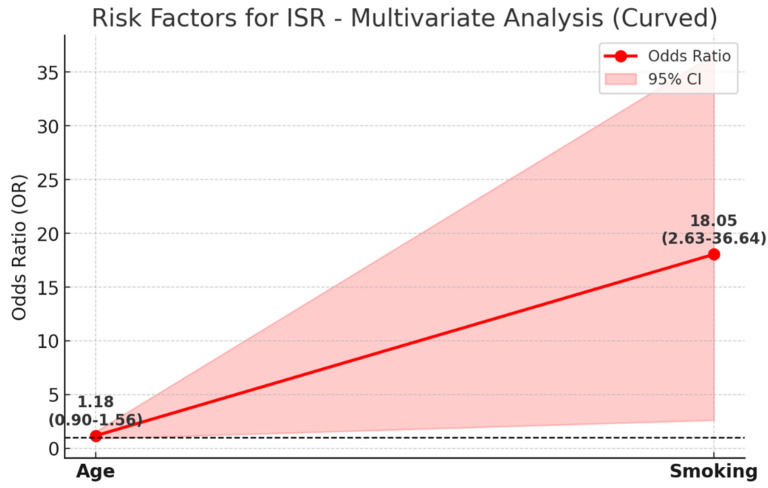
This figure illustrates the multivariate analysis of risk factors for ISR, displaying the odds ratio (OR) values along with their 95% confidence intervals (CI). The red line represents OR values for each risk factor, while the shaded area indicates the corresponding 95% CI. The black dashed line at OR = 1 serves as a reference point, representing no increased risk. Smoking is identified as a significant risk factor for ISR (OR: 18.05; CI: 2.63–36.64), with an 18-fold higher risk in smokers. The wide confidence interval reflects variability, but the *p*-value (0.006) confirms statistical significance. In contrast, age is a weaker risk factor (OR: 1.18; CI: 0.90–1.56), showing a mild increase in ISR risk with aging. However, its confidence interval includes 1, indicating lower statistical significance. This figure highlights the strong association between smoking and ISR compared to the relatively minor influence of age.

Multivariate analysis identified smoking as a significant risk factor for ISR, with an odds ratio (OR) of 18.05 (95% CI: 2.63–36.64), indicating a strong association with restenosis development. In contrast, age showed a weaker and statistically insignificant association with ISR, with an OR of 1.18 (95% CI: 0.90–1.56). The wide confidence interval for smoking suggests some variability, but the overall trend strongly supports its role as a major contributor to ISR risk (Figure 3).

These findings emphasize the critical need for smoking cessation strategies in patients undergoing stenting procedures to reduce the likelihood of restenosis.

## 4. Discussion

In this single-center retrospective study, we evaluated the outcomes of CAS in octogenarians, focusing on restenosis rates, associated risk factors, and post-procedural complications. Our findings indicate that while CAS remains a viable treatment option for elderly patients with carotid artery disease, the incidence of restenosis and periprocedural complications is higher compared to younger cohorts reported in the literature [14,15]. Advanced age, along with comorbid conditions such as hypertension, diabetes mellitus, and chronic kidney disease, appeared to contribute to an increased risk of adverse events. Additionally, anatomical challenges such as increased vessel tortuosity and calcification may have influenced procedural success and long-term patency rates. These results highlight the need for careful patient selection and tailored perioperative management strategies to optimize outcomes in this high-risk population.

Carotid stenting has emerged as a reliable alternative to medical treatment in patients over 80 years of age, with low complication rates observed in our study. Minor complications occurred in 5.3% of cases, with no major complications, supporting its use in high-risk elderly individuals. However, a 12.5% restenosis rate at six months, associated with advanced age, smoking, and initial stenosis severity, highlights the need for careful risk evaluation. Notably, three patients with restenosis successfully underwent re-stenting, demonstrating the method’s applicability for recurrent lesions.

Trials comparing CEA and stenting have emphasized the relatively higher complication rates in elderly patients. The stroke and mortality rate in patients over 80 years of age has been reported as 12%, with minor complications accounting for a significant portion of this figure [16]. This underscores the difficulties associated with CAS in very elderly patients at that time. However, more recent analyses have shown further reductions in minor complication rates. For instance, an observational study published in 2017 reported a 5.6% complication rate in patients aged 80 years and older undergoing CAS. The study highlighted the role of meticulous preprocedural risk stratification and procedural expertise in achieving these improved outcomes [17]. The minor complication rate in our study closely aligns with findings from other significant studies [6]. Collectively, our results and previous studies underscore the need for thorough preprocedural evaluation and multidisciplinary approaches to optimize CAS outcomes in elderly patients.

Restenosis is defined as a narrowing of the blood vessel diameter by more than 50% compared to the initial value. In our study, restenosis occurred in 12.5% of patients during follow-up. Restenosis develops through two main processes: neointimal hyperplasia and vascular remodeling. Neointimal hyperplasia refers to the thickening of the intima due to endothelial cell damage, while vascular remodeling involves structural changes in the affected blood vessel [18]. Endothelial cell damage during surgery or interventional treatment accelerates neointimal hyperplasia by triggering inflammatory mediator activity, a process that may be further intensified by external factors [18,19,20,21]. A meta-analysis reported that the incidence of restenosis after CAS ranged between 10% and 20% [22], consistent with our findings. Additionally, older age, smoking, and stenosis severity were significantly associated with restenosis in our study. Several studies with similar findings have identified age and smoking as independent predictors of restenosis [14,23]. Smoking has been recognized as a key risk factor for carotid atherosclerosis [24], contributing to oxidative stress, vascular inflammation, platelet coagulation, vascular dysfunction, and altered serum lipid profiles, all of which negatively affect the cardiovascular system [25].

Despite its strengths, this study has several limitations. As a single-center retrospective analysis with a relatively small sample size, our findings may not be generalizable to broader populations. Additionally, the six-month follow-up period is relatively short, limiting our ability to assess long-term restenosis and stroke recurrence. Future studies should incorporate longer follow-up periods to better understand the durability of CAS in octogenarians. Furthermore, our study did not include a direct comparison to CEA, which remains a standard treatment for carotid stenosis. A comparative analysis of CAS and CEA outcomes in elderly patients would provide valuable insights into optimal treatment strategies.

Future research should focus on several key areas to further understand the outcomes of CAS in octogenarians. Long-term follow-up studies are needed to assess the durability of CAS and the progression of in-stent restenosis over time. Comparative studies evaluating CAS versus CEA in this age group could provide valuable insights into the relative benefits and risks of each approach. Additionally, personalized risk stratification models incorporating frailty indices, comorbidities, and inflammatory markers may help refine patient selection criteria and predict restenosis risk more accurately. Further research should also investigate the impact of optimized medical management, including antiplatelet therapy, lipid-lowering agents, and lifestyle modifications, on restenosis prevention and overall procedural success. Lastly, studies focusing on patient-reported outcomes, neurocognitive function, and quality of life after CAS in octogenarians could provide a more comprehensive understanding of the long-term impact of the procedure beyond restenosis and complications. Addressing these areas will help improve treatment strategies and optimize outcomes for elderly patients undergoing CAS.

## 5. Conclusions

This single-center study demonstrates that CAS is a viable and effective option for symptomatic carotid stenosis in octogenarians. Despite favorable short- and mid-term outcomes, restenosis and procedural complications remain concerns, particularly in patients with comorbidities and challenging anatomies. Addressing risk factors such as smoking, optimizing patient selection, and employing aggressive medical management strategies may enhance outcomes. Larger, long-term studies comparing CAS and CEA in elderly populations are needed to confirm these findings and refine treatment approaches for high-risk patients.

## Figures and Tables

**Table 1:** This table presents data from 56 stent placement patients (mean age, 85.3 ± 4.40 years; 57.1% male). Common comorbidities included TIA (62.5%), hypertension (55.4%), and diabetes (44.6%). The procedure had a 100% success rate, with 57.1% having severe stenosis. Closed-cell stents were used in 66.1% of cases, and the femoral approach was preferred (87.5%). No major strokes or deaths occurred, with minimal complications (1.7% minor stroke, 1.7% pseudoaneurysm, 1.7% wound hematoma). The findings indicate high success with low risk.

Patient Characteristics and Stent Placement Data	
Characteristic	Value, Number (%)
Age, years, mean ± SD	85.3 ± 4.40
Sex, male	
Male	32 (57.1)
Female	24 (42.9)
Medical history and comorbidities	
History of TIA	35 (62.5)
History of cerebral infarction	21 (37.5)
Hypertension	31 (55.4)
Diabetes mellitus	25 (44.6)
Hyperlipidemia	29 (51.8)
Smoking	17 (30.3)
Procedural Success	56 (100)
mRS score	
0	22 (39.3)
1	25 (44.6)
2	9 (16.1)
Degree of stenosis	
Moderate	24 (42.9)
Severe	32 (57.1)
Stent type	
Open-cell stent	19 (33.9)
Closed-cell stent	37 (66.1)
Access Side	
Femoral	49 (87.5)
Radial	7 (21.5)
Contralateral carotid stenosis	10 (17.9)
Restenosis	7 (12.5)
Type 3 arch	9 (16.1)
Major stroke	0 (0)
Death	0 (0)
Minor stroke, n (%)	1 (1.7)
MI	0 (0)
Pseudoaneurysm	1 (1.7)
Wound hematoma	1 (1.7)

**Table 2:** Baseline characteristics of in-stent restenosis show that patients with restenosis were significantly older (*p* = 0.045). Smoking was more frequent in the restenosis group (*p* = 0.002). Severe stenosis was present in all restenosis cases, showing a significant association (*p* = 0.016). No significant differences were observed in sex, history of TIA or cerebral infarction, comorbidities, or access. * statistically significant, *p* < 0.05.

Baseline Characteristics of In-Stent Restenosis			
Variable	No In-Stent Restenosis (n: 49)	In-Stent Restenosis (n: 7)	*p*-Value
Age, years, mean ± SD	84.8 ± 4.2	88.2 ± 4.7	0.045 *
Sex, n (%)			
Male	29 (59.2)	3 (42.9)	0.447
Female	20 (40.8)	4 (57.1)	
History of TIA, n (%)	32 (65.3)	3 (42.9)	0.406
History of cerebral infarction, n (%)	17 (34.7)	4 (57.1)	0.406
Hypertension, n (%)	25 (51.0)	6 (85.7)	0.116
Diabetes mellitus, n(%)	21 (42.9)	4 (57.1)	0.688
Hyperlipidemia, n (%)	24 (49.0)	5 (71.1)	0.424
Smoking, n (%)	11 (22.4)	6 (85.7)	0.002 *
mRS score, n (%)			
0	21 (42.9)	1 (14.3)	0.312
1	21 (42.9)	4 (57.1)	
2	7 (14.3)	2 (28.6)	
Degree of stenosis, n (%)			
Moderate	24 (49.0)	0 (0)	0.016 *
Severe	25 (51.0)	7 (100)	
Access Side, n (%)			
Femoral	42 (85.7)	7 (100)	0.578
Radial	7 (14.3)	0 (0)	
Type 3 arch	9 (18.4)	0 (0)	0.583
Contralateral carotid stenosis, n (%)	7 (14.3)	3 (42.9)	0.099
Stent type, n (%)			
Open-cell stent	33 (67.3)	4 (57.1)	0.679
Closed-cell stent	16 (32.7)	3 (42.9)	

**Table 3:** This table presents the course of in-stent restenosis retreatment for seven cases. It includes patient age, sex, ISR grade (percentage of restenosis), previous stent type, retreatment method, and complications. ISR grades range from 50% to 80%. Open-cell and closed-cell stents were previously used. Retreatment methods included BMT and CAS. No complications were reported in any case. BMT: best medical therapy; CAS: carotid artery stenting.

Course of In-Stent Restenosis Retreatment					
Case	Age/Sex	ISR Grade	Previous Stent Type	Retreatment	Complication
1	84/M	50%	open-cell	BMT	None
2	92/F	50%	closed-cell	BMT	None
3	91/M	70–80%	open-cell	CAS	None
4	82/F	70%	open-cell	CAS	None
5	94/F	50–60%	closed-cell	BMT	None
6	91/F	50%	open-cell	BMT	None
7	84/M	70%	closed-cell	CAS	None

## Data Availability

The data presented in this study are available on request from the corresponding author.
